# Hybrid Sensor Device for Simultaneous Surface Plasmon Resonance and Surface Acoustic Wave Measurements

**DOI:** 10.3390/s20216177

**Published:** 2020-10-29

**Authors:** Anastasios G. Samarentsis, Alexandros K. Pantazis, Achilleas Tsortos, Jean-Michel Friedt, Electra Gizeli

**Affiliations:** 1 Institute of Molecular Biology & Biotechnology, FO.R.T.H, Vassilika Vouton, 70013 Heraklion, Greece; grad805@edu.biology.uoc.gr (A.G.S.); atsortos@imbb.forth.gr (A.T.); 2 Department of Biology, University of Crete, Vassilika Vouton, 71409 Heraklion, Greece; alepa@physics.uoc.gr; 3 Institute of Electronic Structure & Laser, FO.R.T.H, Vassilika Vouton, 71409 Heraklion, Greece; 4 SENSeOR SAS, Time and Frequency Department, FEMTO-ST Institute, 15B Avenue des Montboucons, 25030 Besançon, France; jmfriedt@femto-st.fr

**Keywords:** biosensors, surface plasmon resonance, surface acoustic wave, Love wave, combined SPR/LW-SAW biosensing platform, 3D printing, PDMS microfluidics, protein adsorption

## Abstract

Surface plasmon resonance (SPR) and Love wave (LW) surface acoustic wave (SAW) sensors have been established as reliable biosensing technologies for label-free, real-time monitoring of biomolecular interactions. This work reports the development of a combined SPR/LW-SAW platform to facilitate simultaneous optical and acoustic measurements for the investigation of biomolecules binding on a single surface. The system’s output provides recordings of two acoustic parameters, phase and amplitude of a Love wave, synchronized with SPR readings. We present the design and manufacturing of a novel experimental set-up employing, in addition to the SPR/LW-SAW device, a 3D-printed plastic holder combined with a PDMS microfluidic cell so that the platform can be used in a flow-through mode. The system was evaluated in a systematic study of the optical and acoustic responses for different surface perturbations, i.e., rigid mass loading (Au deposition), pure viscous loading (glycerol and sucrose solutions) and protein adsorption (BSA). Our results provide the theoretical and experimental basis for future application of the combined system to other biochemical and biophysical studies.

## 1. Introduction

The field of biosensing is closely related to the development of sensitive analytical techniques. Of the available detection methods, optical, such as surface plasmon resonance (SPR) or ellipsometry, and acoustic, e.g., the quartz crystal microbalance (QCM) and surface acoustic wave (SAW) devices, techniques fulfill the basic requirements for label-free and real-time, in-situ measurement of binding processes [1]. Even though both are exploited for the investigation of biomolecular surface-binding events and detection of target-analytes, each one provides complementary information related to the mass, film thickness, viscoelastic properties [1] and conformation [2] of attached biological layers or biomolecules. Combining optical and acoustic sensor technology into a single set-up is, therefore, advantageous for simultaneous monitoring of several parameters and subsequent determination of adsorbed layer/biomolecule physical properties. While studies can be performed on two separate surfaces and then the acoustic and optical results compared, this approach is limited by variations between the two surfaces and the fluidic set-up; it is also more laborious and requires larger amounts of reagents.

For this reason, several combined acoustic and optical set-ups have been developed and reported during the last years. QCM was combined on a single device with SPR technology for the extraction of multiple physical properties of adsorbed layers [3,4,5,6,7,8,9,10,11]. In addition, the combination of QCM with spectrometric ellipsometry has gained much attention lately due to the development and availability of a commercial set-up and tools for modeling the surface mass density and/or thickness of the adsorbed layers [12].

**Figure 1:**
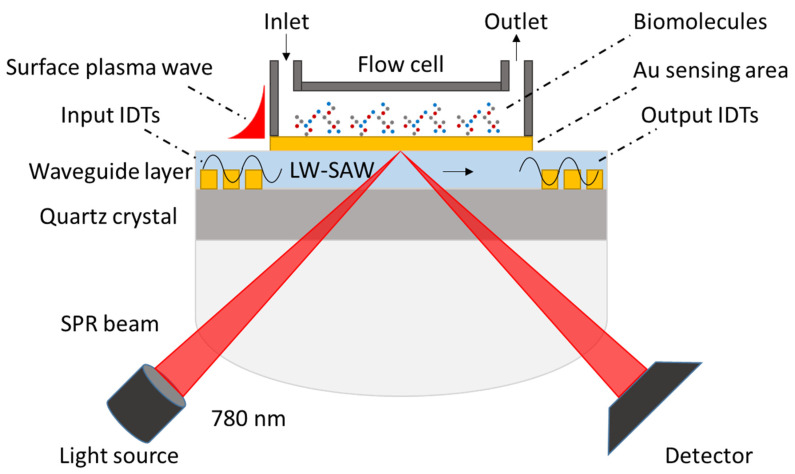
SPR/LW-SAW sensor device (not drawn to scale): A set of electrodes (IDTs) deposited on the quartz surface is used to generate and detect the SAW. A transparent polymer layer acts as a waveguide layer. The optical beam reaches the gold surface from the opposite site and creates an SPR response.

SPR and SAW devices also allow for combined studies, although no commercial system is yet available. In the case of SAW, a Love wave SAW-based (LW-SAW) geometry is normally used for enhanced acoustic response, since the presence of a waveguide layer covering the metallized acoustic surface results in better acoustic wave propagation on the sensing surface and higher sensitivity to surface mass binding events [13,14,15]. From a technical point of view, combining these two technologies is feasible by exploiting the geometrical structure of the SAW device, which leaves the rear of the piezoelectric element free for a light beam to propagate and to produce surface plasmons on a gold coated sensing area (Figure 1).

Based on the above, a set-up for simultaneous SPR/LW-SAW measurements was developed and used to study the thickness and protein-to-water content ratio of several protein layers [16,17]. To incorporate energy losses, the same group employed the quartz crystal microbalance with dissipation monitoring (QCM-D) and succeeded in obtaining information on the conformation of human immunoglobulin protein G (hIgG) [18]. Experimental results were also combined with simulations of an equivalent viscoelastic transmission line model to assess quantitatively the density, viscosity and thickness of adsorbed soft layers [19]. In another study, a combined SPR/LW-SAW system was used for the investigation of the adsorption of neutravidin followed by biotinylated-DNA binding [20]. However, all the above studies came along with two limitations: the use of a static open liquid cell and/or the inability of collecting acoustic energy data.

Here, we present a combined optical (SPR) and acoustic (SAW-Love mode) system for the investigation of biomolecules binding on the device surface based on a flow-through cell and a geometry that allows the simultaneous monitoring of three parameters: acoustic wave velocity and energy and reflected light intensity (Figure 1). To achieve the above, we first investigated the experimental parameters required to match simultaneously the conditions for efficient SPR coupling and acoustic waveguiding. Moreover, a fluidic system consisting of a 3D-printed plastic holder integrated with a PDMS microfluidic chip was designed and fabricated to allow experiments under continuous flow. The optical and acoustic responses were calibrated with respect to glycerol/water and sucrose/water solutions. Finally, the system was used to study BSA adsorption on gold.

## 2. Materials and Methods

### 2.1. SPR/LW-SAW Sensor Device

**Figure 2:**
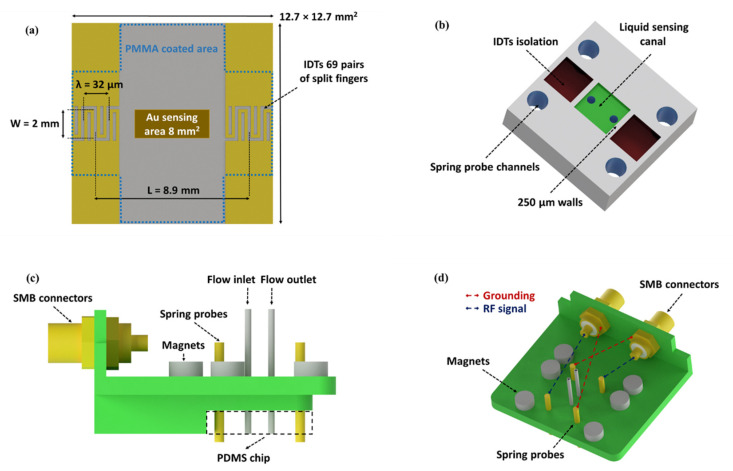
(**a**) SPR/LW-SAW sensor device; (**b**) PDMS microfluidic chip; (**c**) 3D-printed holder (side view); and (**d**) 3D-printed holder (top view).

The SPR/LW-SAW sensor devices were designed and fabricated in-house. The fabrication process was performed using photolithography. ST-cut quartz substrates (Y-cut 42°45’), 500 μm thick, were employed in a single delay line structure (Roditi International Corporation Ltd., London, UK). The photolithographic mask was designed using CleWin V.3 software (PhoeniX BV, Hengelo, The Netherlands) and it was manufactured by JD Photo Data (Hitchin, UK). Conventional contact UV lithography, e-beam metal deposition, metal lift-off and wafer dicing were applied in a two-step fabrication procedure. AZ 5214 photoresist (MicroChem, Westborough, MA, USA) was used for the photolithographic process, along with an MA6/BA6 mask-aligner from SUSS MicroTec (Garching, Germany). The first step included a metal deposition, by an e-beam BJD 1800 evaporator from Temescal (Livermore, CA, USA), of 10 nm Cr/200 nm Au and lift-off process in order to fabricate the IDTs and contact pads. In the second step, a mechanical wheel was used in order to dice the processed wafer into chips. The dimensions of the device were 12.7 mm × 12.7 mm. Each IDT structure consists of 69 split fingers with a wavelength of 32 μm, which corresponds to a theoretical frequency of operation of 154.75 MHz. Fingers width and spacings were 4 μm, each IDT being 2.1 mm in length, with an aperture of 2 mm, and the IDT center-to-center distance was 8.9 mm (Figure 2a).

### 2.2. PMMA Guiding Layer 

Polymer guiding layers made of PMMA (MicroChem, Westborough, MA, USA) solutions were deposited on the surface of the acoustic device by spin coating (P-6708D spin coater model, SCS, Indianapolis, IN, USA). We tested two different PMMA products with 495 and 950 kDa molecular weight (MW) resins at various concentrations, dissolved in anisole. The PMMA solutions were spin coated at speeds from 1000 to 4000 rpm, to produce a wide range of thicknesses. The spin coating time was stable at 45 s and the devices were post-baked using a hotplate at 180 °C for 90 s, to cross-link the polymer. The PMMA covers the area over the IDTs and the delay path, while the pads were protected using adhesive tape (Figure 2a).

### 2.3. Au Sensing Area Sputter Coating

A layer of Au was sputter coated on the top of the PMMA guiding layer as the sensing area. The Au layer is necessary for the excitation of surface plasmons and it can promote the physical adsorption of proteins. Layers of different thicknesses were deposited in order to investigate the effect on the SPR coupling and the acoustic response. This procedure was performed with a Bal-Tec SCD 050 sputter coater (Bal-Tec Balzers, Liechtenstein). For the conversion of the sputter coating time to Au layer thickness, calibration studies were performed by measuring the thickness after deposition via Field Emission Scanning Electron Microscopy (FESEM). To directly relate the signal change to the deposited mass, we used the thickness calibration factor and the Au mass density (19.3 g/cm^3^).

### 2.4. 3D-Printed Holder

A device holder was designed on computer-aided design (CAD) software and fabricated via 3D printing. The holder was made from PolyPlus or PolyLite polylactic acid (PLA) (Polymaker, Shanghai, China) using a LulzBot TAZ 6 desktop 3D printer (Aleph Objects Inc., Loveland, CO, USA). The dimensions of the holder were dictated by the mount-system of the SPR instrument (Figure 2c,d). The main role of the holder is to electrically connect the sensor device with the network analyzer (NA). The design embodied an area for the placement of a PDMS microfluidic chip in order to assemble a flow-through system. A crucial parameter to consider is the pressure applied on the device surface which affects the acoustic signal [21]. The specific holder incorporates miniaturized magnets (Magnitech, Athens, Greece), which allow a standard pressure to be applied to the device surface by the flow cell for each experiment. For this purpose, six magnets, with diameter and thickness of 4 mm, were used and attached to the plastic holder using epoxy glue. SMB connectors and spring probes were purchased from RS Components (Corby, UK).

### 2.5. PDMS Microfluidics

A PDMS microfluidic chip was developed to build a closed channel which enables to work in a liquid environment (Figure 2b). Walls, 250 μm thick, were used to restrain the liquid in the sensing area and protect the IDTs. Two air cavities were used above the IDTs to avoid wave attenuation. The PDMS microfluidic fabrication process was based on an SU-8 photoresist master mold. The master mold was made on a 4-inch Si wafer (Siegert wafer GmbH, Aachen, Germany) by photolithography. PDMS (Sylgard 184 Dow Corning) was purchased from Ellsworth Adhesives Europe (East Kilbride, Scotland). A 10:1 mixture of PDMS (40 g Silicone agent:4 g curing agent) was poured into the mold and placed in a vacuum desiccator to eliminate air bubbles. After the degassing step, the mold was placed in an oven at 60 °C for 2 h. Upon baking, the PDMS replica was taken off the mold. To allow injection of fluids, inlet and outlet holes were punched using 0.7 mm PDMS punchers (World Precision Instruments Inc., Sarasota, FL, USA).

### 2.6. Assembly and Instrumentation

**Figure 3:**
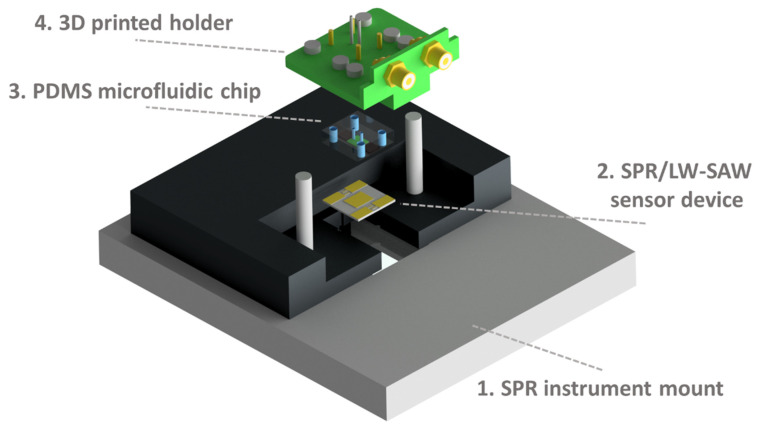
Experimental set-up assembly: First, the SPR/LW-SAW sensor device (2) is placed on the mount of the SPR instrument (1). Then, the PDMS chip (3) is fixed to the 3D-printed holder (4) and applied on the device surface.

In the present study, the SPR measurements were performed using a modified commercial instrument, the SR7000 SPR single-channel refractometer by Reichert Technologies (Depew, NY, USA), which employs a Kretschmann ATR configuration [22]. The instrument comprises a 780 nm laser diode as the light source and a 3696-pixel linear CCD array for the detection of the reflected light [23]. A Hewlett Packard 8753ES network analyzer (Keysight Technologies, Santa Rosa, CA, USA) was used to generate and detect the acoustic wave by monitoring the acoustic velocity (phase) and energy (amplitude) changes. The gating function of the network analyzer was used for removing unwanted responses/noise in the time domain, resulting in the smoothing of both of the recorded acoustic signals. Data were collected using a dedicated SPR/LW-SAW control and acquisition software, which was based on Python programming also allowing for graphics display (PSF, Wilmington, DE, USA). Initially, the sensor device is placed on the SPR mount and subsequently the PDMS chip is assembled with the 3D-printed holder (Figure 3). The holder is electrically connected to the NA and mechanically to a peristaltic pump for the initiation of the flow. The excitation of surface plasmons occurs from the opposite side of the sensor device through a polarized laser beam.

### 2.7. SPR/LW-SAW Device for Liquid Studies 

The sensor device was prepared prior to experiment, i.e., a PMMA layer was spin-coated and an Au layer was sputter coated. At the end of experiment, the Au sensing area was removed using ethanol; for the next measurement, a new Au layer was deposited. The acoustic response was recorded after every step (bare device, PMMA and Au deposition) to confirm reproducibility. All measurements were conducted under continuous flow conditions with a flow rate of 20 µL/min using a Gilson Miniplus 3 peristaltic pump (Gilson, Middleton, WI, USA). At the beginning, the device was kept under phosphate-buffered saline (PBS) (Sigma-Aldrich, St. Louis, MO, USA) or d.i. H_2_O flow to reach a stable baseline. After the end of experiment, the sensing area and the tubing were cleaned by 2% Hellmanex II (Fischer Scientific, Loughborough, UK) aqueous solution. All experiments were performed at 24 ± 1 °C. Glycerol solutions in water were prepared at various concentrations using anhydrous pure glycerol stock obtained from AppliChem (Darmstadt, Germany). Water/sucrose solutions were prepared using sucrose at powder form obtained from Merck Millipore (Burlington, MA, USA). BSA (Sigma-Aldrich, St. Louis, USA) solutions were prepared in PBS (pH 7.4).

## 3. Results and Discussion

For the development of a hybrid SPR/LW-SAW wave device, the two geometries should be optimized separately and then combined for parallel optical and acoustic measurements. Regarding the latter, parameters of interest are the linearity of the phase signal, the maximum value of the amplitude and the amplitude vs. frequency envelope shape. The above should be examined as a function of the waveguide layer thickness and the applied pressure of the flow cell on the SAW device, both known to affect the quality of the acoustic signal [21].

### 3.1. Love-Wave Device Optimization

**Figure 4:**
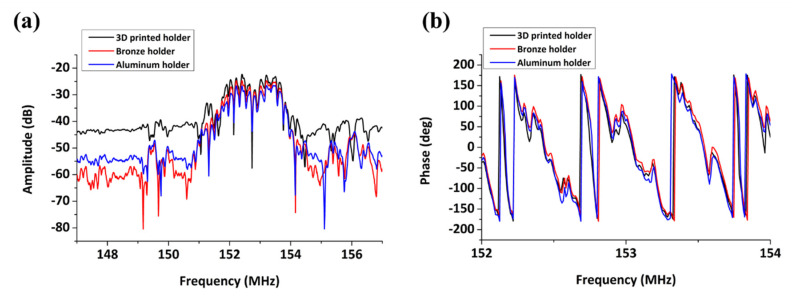
Uncoated device in air: (**a**) amplitude spectrum; and (**b**) phase spectrum.

Initially, the effect of the 3D-printed device holder to the amplitude and phase signals was tested, with respect to its ability to shield equally well with the metal holders from external electromagnetic effects. In Figure 4, the acoustic amplitude and phase spectrum of uncoated SAW device is presented when a bronze, aluminum or 3D-printed device holder is applied on the sensor.

According to this figure, while the acoustic spectrum is similar around the central frequency (152.4 MHz), the background noise level depends on the holder material with the bronze holder providing the highest peak amplitude-to-background noise difference (−35.5 dB). However, in all cases, the resulting signal level is sufficient for acoustic measurements (Al: −28.8 dB, 3D: −22.3 dB). The presence of ripples suggests strong reflections of the wave coming from the edges and/or the rear surface of the device, independently of the type of device holder used. Upon liquid loading, the bare devices do not exhibit any considerable amplitude drop. Based on the above, the 3D-printed holder was chosen since it allows for lab-based, inexpensive and fast prototyping without the need for a micro-machine workshop.

**Figure 5:**
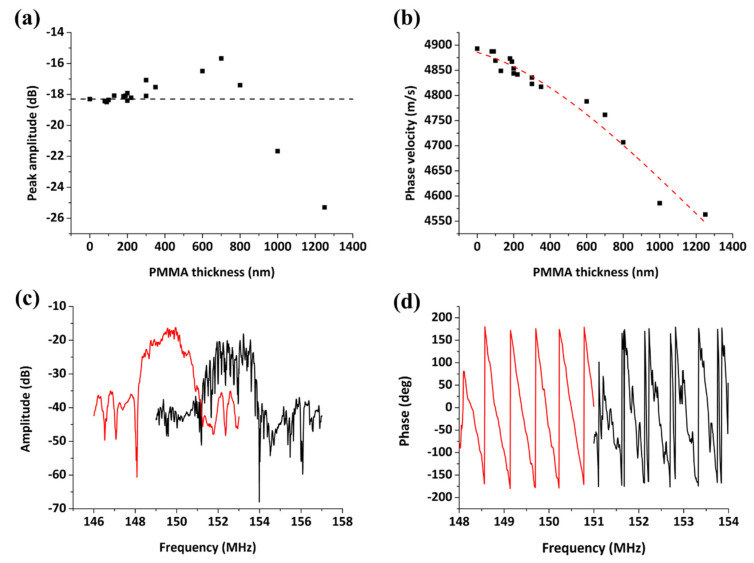
Peak amplitude (**a**) and phase velocity (**b**) shift as a function of the PMMA guiding layer thickness, uncoated versus PMMA-coated device (700 nm) in air: (**c**) amplitude and (**d**) phase spectrum.

Ripples appearing on the spectra in Figure 4 can be attributed to the fact that ST-cut quartz substrates initially excite surface skimming bulk waves (SSBW) which suffer from beam spreading loss inside the bulk of the crystal [24]. Deposition of a polymer or silicon dioxide over-layer converts the SSBW to Love wave, which can be used efficiently for liquid phase detection. An experimental approach is normally used to determine the optimum thickness, i.e., the one that provides both efficient waveguiding with tolerable loss upon liquid loading and high sensitivity in biomolecule detection. As shown in Figure 5a, the signal strength (peak amplitude) is increasing up to a PMMA thickness of 700 nm, while a decrease in phase velocity is observed with increasing PMMA thickness (Figure 5b), consistent with previous studies [25,26,27]. Hence, for our sensor design, a PMMA layer of ~700 nm thickness was found to provide the minimum insertion loss combined with a smooth acoustic spectrum envelope and a linear phase-to-frequency response (Figure 5c,d).

### 3.2. Au Layer Optimization for a Combined SPR/LW-SAW Geometry

The hybrid sensor geometry should incorporate a thin metal film deposited on the sensor surface necessary for the excitation of surface plasmons, typically a layer of Au with thickness around 50 nm [28]. In our study, Au films of various thicknesses were deposited via sputter-coating to the sensor surface. This provides information about the SPR reflectivity profile as a function of the Au layer thickness, but also allows calibrating the LW-SAW response to rigid surface mass loading [29,30,31].

**Figure 6:**
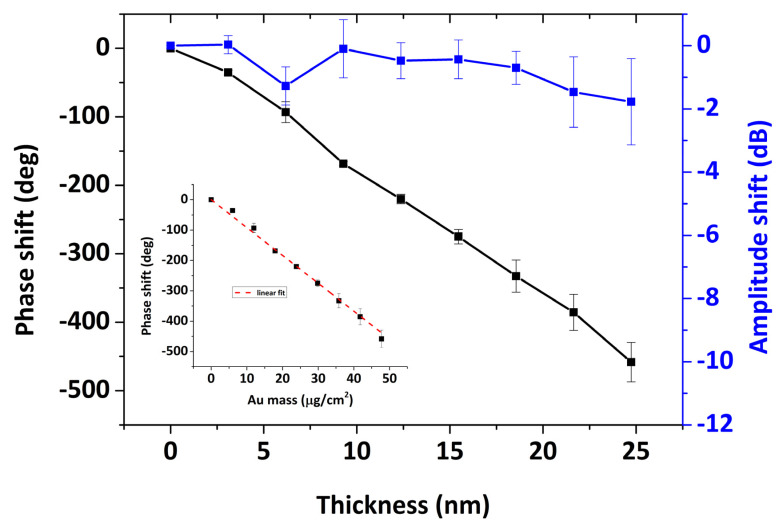
Phase and amplitude shift upon Au deposition up to 24 nm on the sensor surface. (**inset**) Average phase shift as a function of Au mass per unit area (dashed line, linear fit, resulting in slope 9.17 deg cm^2^/g).

In Figure 6, the amplitude and phase shifts are presented for Au deposition of thicknesses up to 25 nm for the SPR/LW-SAW devices. For rigid mass loading the change in acoustic wave amplitude is expected to be close to zero while the phase change to be proportional to the deposited mass [32,33]. In the Figure 6 inset, the slope value corresponds to 9.17 deg cm^2^/μg. If the effective area is determined as only the metallized section of the wavepath, using this slope, we get an estimation of the phase sensitivity to surface mass for rigid loading $S_{\Phi }$ which corresponds to

(1) $$S_{\Phi }=\frac{\Delta \varphi }{\varphi_{0}}\frac{A}{\Delta m}=163\;\frac{{\mathrm{cm}}^{2}}{g}$$

where $\Delta m=\rho_{Au}\times h\times A$ is the deposited mass, $A=0.5\times 0.2\;{\mathrm{cm}}^{2}$ is the sensing area, $\rho_{Au}=19.3\;g/{\mathrm{cm}}^{3}$ is the Au mass density, h is the layer thickness, Δφ (°) is the phase signal change upon deposition and $\varphi_{0}=360{}^{\circ}\times \left(x/\lambda \right)=56250{}^{\circ}$ is the unperturbed phase for the effective length x=0.5 cm. The above estimated gravimetric sensitivity matches quite well the sensitivity values obtained in previous SPR/LW-SAW studies, using copper electrodeposition on silicon dioxide guiding layers as the calibration technique $\left(145-165\;{\mathrm{cm}}^{2}/g\right)$ [16,17].

**Figure 7:**
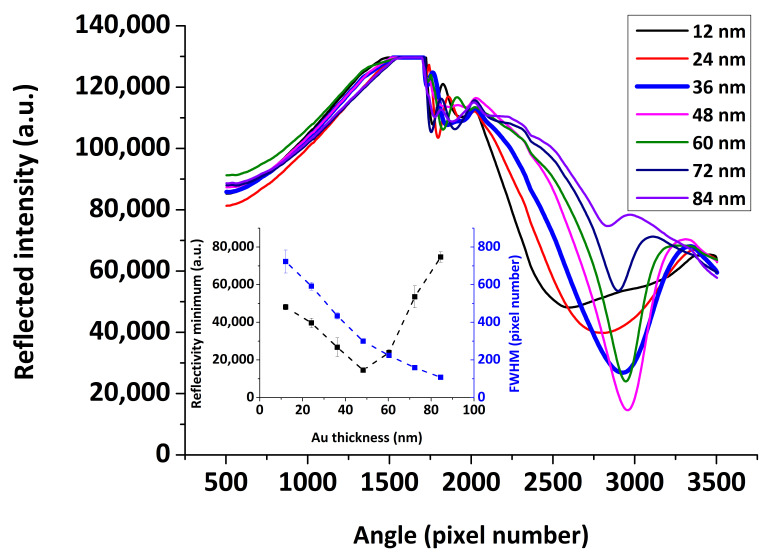
SPR reflectivity curves versus angle pixel number for various Au layer thicknesses. (**inset**) FWHM and minimum reflectivity as a function of the Au thickness.

**Figure 8:**
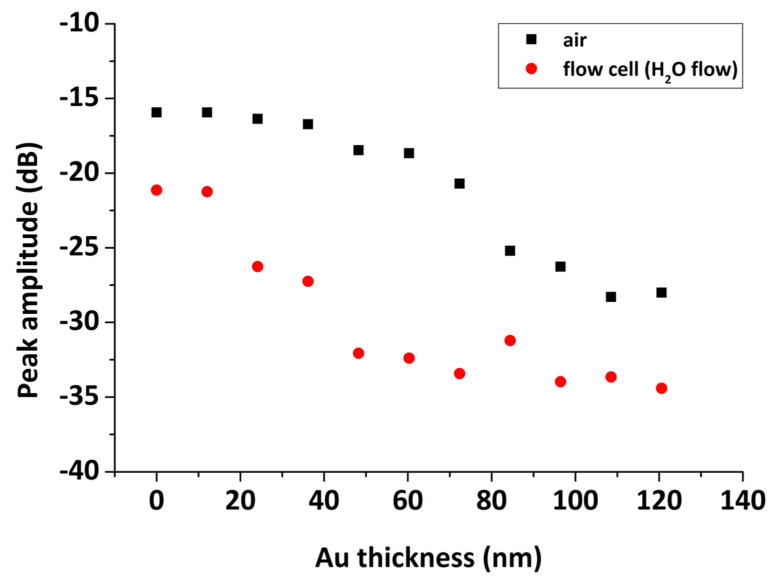
Peak amplitude versus Au layer thickness up to 120 nm, deposited on the PMMA-coated sensor surface, in air and in H_2_O flow.

The effect of the Au layer thickness on the SPR reflectivity curve using our combined SPR/LW-SAW device is presented in Figure 7, for a range of Au thicknesses of 12–84 nm deposited on the PMMA coated quartz substrate and upon water loading. It is well-known that the thickness of the metal layer has a great impact on the shape of the SPR reflectivity curve [34]. The optimum thickness is the one that provides the sharpest dip in reflected light intensity, which can be quantified in terms of SPR curve key parameters such as full width at half maximum (FWHM) and reflectivity minimum (Figure 7, inset). It was confirmed that the optimum Au thickness is close to 50 nm and that the SPR “dip” becomes shallower above this thickness and broader below. Nonetheless, by increasing the Au layer thickness above 40 nm, the insertion loss of the acoustic device becomes significantly high in the presence of a liquid medium, drops below −30 dB, resulting in acoustic measurements with low reproducibility for our system (Figure 8). Based on the above and as a compromise between an optical and acoustic geometry, we chose to use for the SPR/LW-SAW device an Au sensing layer of 36 nm.

**Figure 9:**
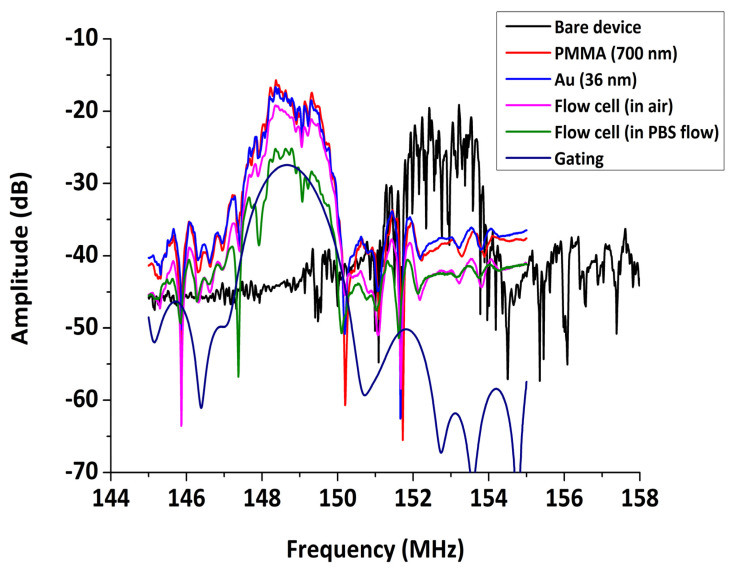
Acoustic transmission spectrum at different stages prior to experiment.

The amplitude spectrum of the sensor device at the stages prior to the experiment is presented in Figure 9. Initially, the uncoated device is characterized by some strong reflections, which are partially eliminated with the deposition of the 700 nm PMMA waveguide layer. The deposition of a 36 nm Au sensing area causes a slight drop of the peak amplitude. The fixing of the flow cell onto the device further increases the insertion loss, while the presence of liquid medium on the sensing area causes an extra decrease of the peak amplitude (−6 dB), which remains however at an acceptable level. At the end, the gating function of the NA was used for smoothing of the signal.

### 3.3. SPR/LW-SAW Calibration (Glycerol/Water and Sucrose/Water Solution Sensing)

To evaluate the combined SPR/LW-SAW set-up, a series of glycerol–water and sucrose–water solutions were applied to the sensor surface. Depending on the glycerol and sucrose concentration, the mixtures correspond to different known values of density, viscosity and refractive index [35]. Hence, these experiments offer an appropriate strategy for the simultaneous examination of the optical and acoustic response of the system. The SPR response measures changes in the bulk index of refraction of solutions in contact with the sensor surface [36,37]. The acoustic responses, amplitude and phase, measure changes of the square root of the density–viscosity product $\sqrt{\rho \eta }$ of solutions upon pure viscous loading on the sensor surface [38,39].

**Figure 10:**
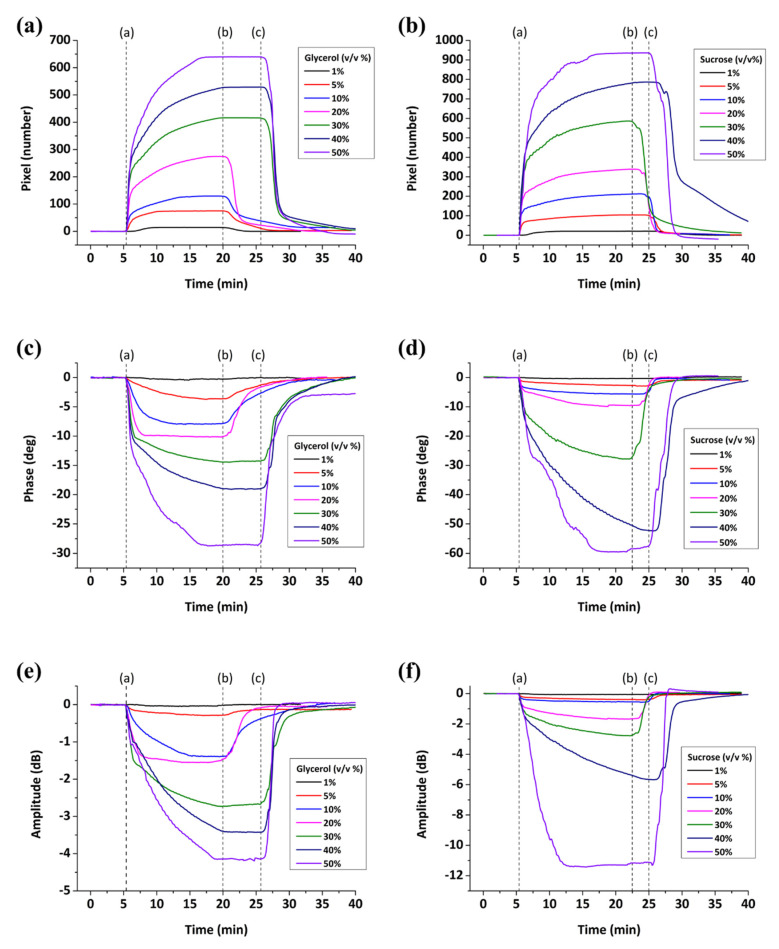
Real-time response upon glycerol/water and sucrose/water solutions loading on the sensor surface: SPR (**a**,**b**); phase (**c**,**d**); and amplitude (**e**,**f**).

Some typical real-time SPR, phase and amplitude measurements are presented in Figure 10. Glycerol and sucrose mixtures were prepared at various concentrations (1–50% v/v) and passed over the sensor surface. The applied solutions provided a range of refractive indices from 1.335 to 1.445 for sucrose/water solutions and from 1.334 to 1.405 for glycerol/water solutions (referring at a wavelength of 589 nm, 20 °C). In terms of viscosity, the sucrose mixtures provided a range from 1.043 to 115.7 cP, while the glycerol mixtures from 1.028 to 8.057 cP (at 20 °C). All graphs exhibit the following steps: at Point (a), water is replaced by glycerol/sucrose solutions until signal saturation, at Point (b) or (c) depending on the concentration, the mixtures are replaced by water. The exchange of pure water with glycerol and sucrose solutions causes an SPR increase while the phase and amplitude signals are decreasing until equilibrium is reached. Upon subsequent water rinse, the reverse effect is observed where the signals return to the initial baseline level.

#### 3.3.1. SPR Calibration 

**Figure 11:**
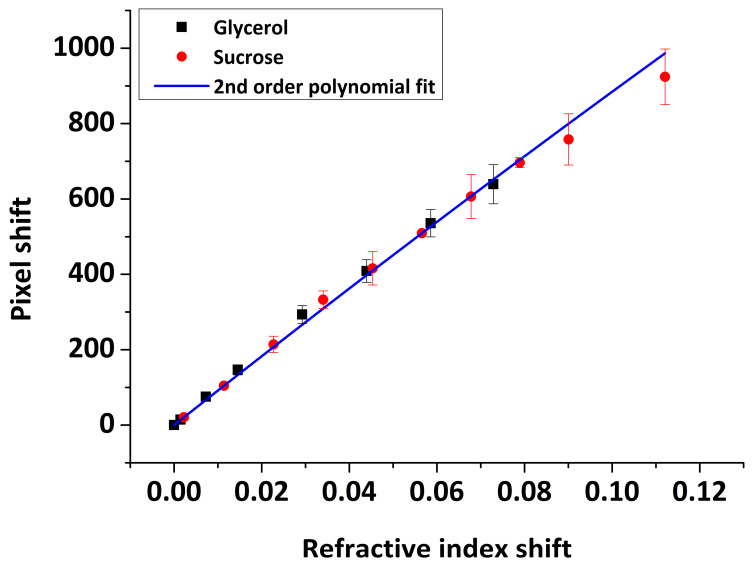
Pixel shift versus normalized (to pure water) refractive index shift for glycerol/sucrose solutions.

The reflected light intensity profile, thus the SPR minimum position and its shift upon optical index changes on the sensor surface, are expressed as a function of the detector element number in pixels. Although the SPR response expressed in pixel units is sufficient to perform kinetic analysis of biomolecular interactions and estimate association and dissociation constants, it cannot be used to estimate the adsorbed mass or the thickness of the adsorbed layer. For this purpose, the SPR output should be converted to resonance angle shift (deg). As a result, a proper calibration of the SPR instrument is required in order to determine a conversion factor between the incident light angle and the pixel number of the detector element of the reflected light. A previously reported calibration method is the use of an SPR goniometer, which mechanically rotates the prism/sensor component against the incident light beam [36]. However, this approach requires additional hardware. The alternative method is to take advantage of the dependence of the SPR minimum position on the bulk refractive index of the medium present on the sensor surface. Following the latter approach, we used a series of glycerol/water and sucrose/water solutions of well-known refractive index values. Using the real-time data, we measured the pixel shift between water and the applied solution. The pixel shift versus the bulk optical index of solutions is presented in Figure 11, normalized to pure water optical index value $n_{w}=1.333$. A second-order polynomial fit was proved more accurate than a linear curve fit, although a linear relationship can be used over a narrow enough optical index range. The result from the SPR instrument measurements was

(2) $$p=9202\;\Delta n-3579\;\Delta n^{2}$$

$\Delta n=n_{s}-n_{w}$, $n_{s}$ is the refractive index of glycerol/sucrose solutions and p is the pixel shift.

The relationship above provides a calibration of the optical response in the manner that the output signal of the SPR instrument (pixel shift) can be translated to optical index changes. However, the SPR angle shifts are reported as pixels on the CCD detector and a conversion factor from pixel to degree is necessary for the quantitative analysis of the optical measurements and the comparison of our data to the literature. By using the relationship obtained experimentally and the theory of SPR, it is possible to find a conversion factor from pixels to degrees.

According to theory, the dispersion relation for surface plasmons propagating along the interface between a metal and a dielectric is given by [34,40,41]

(3) $$k_{sp}=\frac{\omega }{c}\;{\left(\frac{\varepsilon_{\omega }\varepsilon_{\alpha }}{\varepsilon_{\omega }+\varepsilon_{\alpha }}\right)}^{\frac{1}{2}}$$

where $k_{sp}$ is the wavevector of propagating surface plasmons, ω is the angular frequency, c is the speed of light in vacuum,$\;\varepsilon_{\omega }=\varepsilon^{\prime }\left(\omega \right)+i\;\varepsilon^{\prime \prime }\left(\omega \right)$ is the complex dielectric function of metal and $\varepsilon_{\alpha }$ is the dielectric constant of the medium.

For the excitation of surface plasmons, light is guided through a high refractive index prism and it is incident to the metal surface with an angle θ. The wavevector of the light parallel to the surface $k_{x}$ is given by

(4) $$k_{x}=\frac{\omega }{c}\;\varepsilon_{g}{}^{\frac{1}{2}}\sin\theta$$

where $\varepsilon_{g}$ is the dielectric constant of the prism.

By varying the angle of incidence, a minimum in the intensity of the reflected light is observed at the resonance angle $\theta_{sp}$, which corresponds to the optical excitation of surface plasmons. Then, the resonance condition can be expressed by

(5) $$\varepsilon_{g}{}^{1/2}\sin\theta_{sp}={\left(\frac{{\varepsilon^{\prime }}_{\omega }\varepsilon_{a}}{{\varepsilon^{\prime }}_{\omega }+\varepsilon_{a}}\right)}^{\frac{1}{2}},k_{\mathrm{sp}}=k_{x}$$

**Figure 12:**
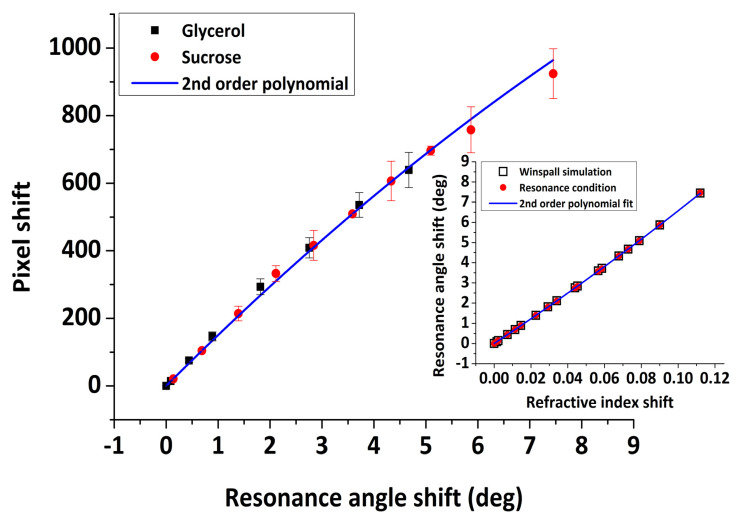
Pixel versus resonance angle shift. (**inset**) Angle shift versus optical index shift using Equation (3) and Winspall software simulations, normalized to water values.

Equation (5) provides a relationship between the dielectric constant of the medium and the resonance angle position. It is a simplified analytical equation that allows assessing the position of the resonance angle which critically depends on the dielectric constant of the medium, assuming a semi-infinite metal film. Nonetheless, it provides a good approximation of the resonance angle position. On the other hand, it cannot give any information about the profile of the resonance curve which depends mainly on the optical properties and the thickness of the metal layer. As in our case, for a multi-layered system, the shape of the SPR curve can be described by Fresnel equations. In this study, we ran simulations using dedicated software developed for this purpose (Winspall software, version 3.0.2 [42]). The fixed optical parameters used in the simulations were the following: Sapphire prism, n=1.7607, k=0; Quartz, n=1.4537, k=0, PMMA, n=1.4851, k=0; and Au, n=0.14737, k=4.7414, taken from [43]. Both the analytical solution from Eq.5 and Winspall simulations resulted in the same relationship (Figure 12 inset) between the resonance angle and optical index shift (normalized)

(6) $$\Delta \theta =60.1\;\Delta n+56.2\;\Delta n^{2}$$

where Δθ(deg) is the resonance angle shift.

Subsequently, by using the experimental relationship between pixel and optical index and the theoretical between resonance angle and optical index, a conversion factor is obtained from pixel shift to resonance angle shift, as presented in Figure 12

(7) $$p=153.6\;\Delta \theta -3.2\;\Delta \theta {\;}^{2}$$

According to a linear fit of the data presented in Figure 11, the SPR sensitivity corresponds to 9006±8 pixel/RIU (i.e., 1 pixel = 111 μRIU, R^2^= 0.9999), compared to a value of 10,034±33 pixel/RIU obtained in previous studies using the same instrument with gold-coated glass slides [23] or 11,493±32 pixel/RIU based on the manufacturer specifications. Considering the resonance angle to pixel relation (Figure 12), one pixel corresponds to 0.007° in comparison to ∼0.005° provided by the manufacturer [23].

#### 3.3.2. LW-SAW Calibration 

For liquids on a LW-SAW device, a shift is caused on both phase and amplitude of the acoustic wave. The measured quantities, amplitude and phase are related to the wave characteristics, i.e., the relative change of the wave velocity and the attenuation per wavenumber [44]. For the viscous loading of a shear SAW device and providing that the liquid is Newtonian, the wave characteristics are proportional to the square root of the density–viscosity product of the liquid $\sqrt{\rho \eta }$ [45]. Hence, we get an expression for the sensitivity upon viscous loading on the sensor surface

(8) $$S_{viscous,Phase}=\frac{\Delta \varphi }{\sqrt{\rho \eta }}$$

(9) $$S_{viscous,Amplitude}=\frac{\Delta A}{\sqrt{\rho \eta }}$$

Furthermore, a linear correlation between phase and amplitude change should be expected for Newtonian liquids, which can be evaluated from Equation (10) [46]:

(10) $$\frac{\Delta \varphi \;\left(\deg\right)}{\Delta A\;\left(\mathrm{dB}\right)}=\frac{360}{40\pi \log\left(e\right)}=6.6\;\deg\;{\mathrm{dB}}^{-1}$$

**Figure 13:**
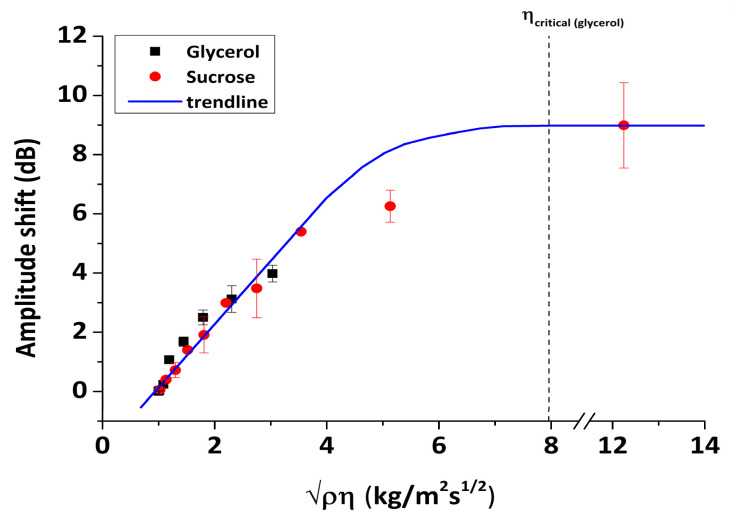
Amplitude response (normalized to water) versus the square root of the density–viscosity product for glycerol/water and sucrose/water solutions.

**Figure 14:**
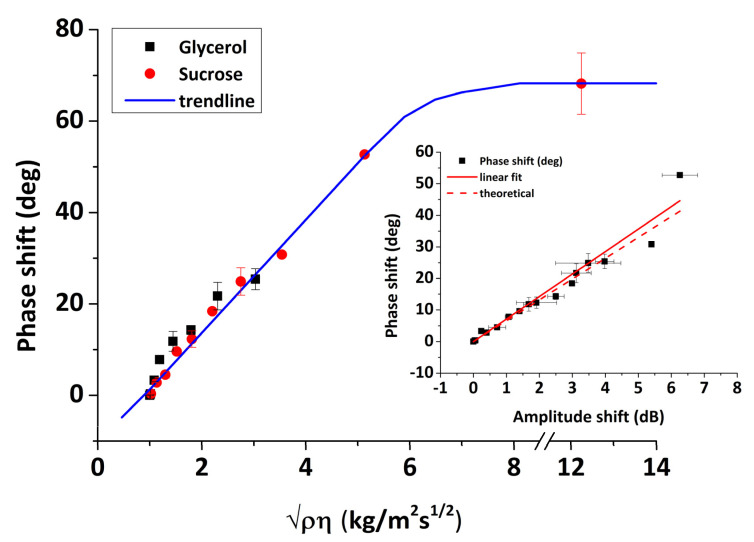
Phase response versus the square root of the density–viscosity product for glycerol/water and sucrose/water solutions. (**inset**) Phase versus amplitude response (solid line, linear fit for the experimental data 7.12 deg/dB; dashed line, theoretical slope 6.6 deg/dB).

As a result, by introducing a series of glycerol and sucrose solutions in water on the sensor surface, we can assess the viscous sensitivity of phase (Equation (8)) and amplitude (Equation (9)) responses and in parallel to confirm the proper response of the acoustic sensor by checking the validity of Equation (10). The values of Δφ (deg) and ΔA (dB) were calculated from the real-time figures (Figure 10) and correspond to the signal difference between water and glycerol/sucrose solutions at saturation. In Figure 13 and Figure 14, the changes in amplitude and phase are presented as a function of $\sqrt{\rho \eta }$, respectively. The data exhibit a linear response for both phase and amplitude as the value of $\sqrt{\rho \eta }$ was increased, indicating a Newtonian response in agreement with previous experimental work [39]. The sensitivities to viscosity measurements were calculated based on the slopes of Figure 13 and Figure 14, leading to the following values: 2.1 dB/kg/(m^2^s^1/2^) and 12.4 deg/kg/(m^2^s^1/2^) for amplitude and phase, respectively. The linear relationship between the acoustic response and the square root of the density–viscosity product of liquids should exist up to a critical viscosity value η_c_, beyond which liquids behave as Maxwellian; for our system, the theoretical glycerol critical viscosity value corresponds to ${\left({\rho \eta }_{c}\right)}^{1/2}=7.96\;\mathrm{kg}m^{-2}s^{-1/2}$ (shown as a vertical dot-line in Figure 13). Unfortunately, sensor instability and the microfluidics employed did not allow for more (accurate) points to be measured above $\sim 4\;\mathrm{kg}m^{-2}s^{-1/2}$. Thus, although a precise value cannot be determined, loss of Newtonian behavior appears to fall in the right range of values (5–8$\;\mathrm{kg}m^{-2}s^{-1/2})$. An additional test against theory is presented in Figure 14 (inset). A linear correlation between phase and amplitude is clearly confirmed for both glycerol and sucrose solutions; here, the best-fit line has a slope of 7.12 deg/dB, in very good agreement with the theoretical value of 6.6 from Equation (10).

### 3.4. BSA Adsorption

**Figure 15:**
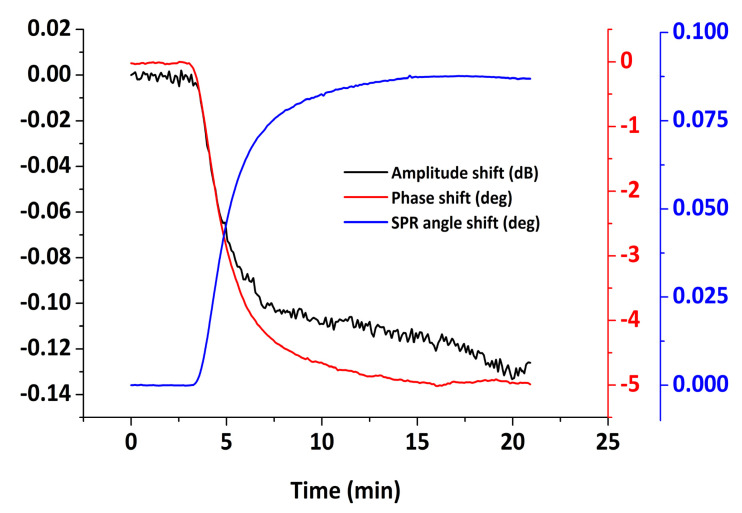
Real-time SPR, phase and amplitude responses upon BSA adsorption on Au.

The combined SPR/LW-SAW system was used to investigate the adsorption behavior of BSA on Au. BSA adsorption has been widely studied with different techniques on various solid surfaces [47,48,49,50,51,52,53]. In this study, BSA was directly adsorbed on the hybrid sensor’s surface at a concentration of 200 μg/mL, with a final sample volume of 500 µL, which is a quantity sufficient to approach a saturation coverage. To establish a stable baseline, PBS (pH 7.4) was flown over the device surface before the introduction of protein. After the adsorption, any unbound protein was removed during a subsequent PBS rinse. The averaged real-time optical and acoustic measurements are presented in Figure 15, extracted from three experiments. The SPR signal shift at saturation and after buffer rinsing is 88 ± 8 mdeg, using the calibration factors obtained in the previous section. On the other hand, the phase and amplitude signal shifts result in 4.9 ± 0.2 deg and 0.11 ± 0.05 dB, respectively.

For the quantitative interpretation of the SPR response, we implemented the mathematical formalism proposed by Jung et al. [37] using the equations

(11) $$\Delta \theta \left(\deg\right)=m_{1}\left(n_{a}-n_{s}\right)\left[1-e^{-\frac{2d}{l_{d}}}\right]+m_{2}{\left\{\left(n_{a}-n_{s}\right)\left[1-e^{-\frac{2d}{l_{d}}}\right]\right\}}^{2}$$

(12) $$l_{d}\left(\mathrm{nm}\right)=\left(\frac{\lambda }{2\pi }\right)/Re{\left\{-n_{eff}{}^{4}/\left(n_{eff}{}^{2}+\varepsilon_{\omega }\right)\right\}}^{1/2}$$

where $m_{1}=60.1\;\mathrm{and}\;m_{2}=56.2$ are calibration factors obtained earlier from glycerol/sucrose solutions (Equation (6)), d (nm) is the optical film thickness and $l_{d}=318\;\mathrm{nm}$ is the decay length estimated from Equation (12) for the effective refractive index of the sample $n_{eff}=1.331+0.00149\approx 1.332$. To obtain the refractive index of BSA, we used the particular amino acid sequence and related optical parameters given in [54], following the procedure employed in [55]. The refractive index increment at 780 nm is given by

(13) $${\left(\frac{dn}{dc}\right)}_{780}={\left(\frac{dn}{dc}\right)}_{578}\times \left(0.940+\frac{20,000}{780^{2}}\right)$$

taking ${\left(dn/dc\right)}_{578}=0.190\;\mathrm{mL}/g$ for BSA [56]. The equation

(14) $${\left(\frac{dn}{dc}\right)}_{780}=\frac{3}{2}\;{\bar{\nu }}_{a}n_{s}\frac{n_{a}^{2}-n_{s}^{2}}{n_{a}^{2}+2n_{s}^{2}}$$

is used to calculate $n_{a}=1.593$ with BSA partial specific volume ${\bar{\nu }}_{a}=0.7347$ cm^3^/g estimated from the sequence [55]. The refractive index of PBS buffer at 780 nm was estimated from the Cauchy parameters given in [57] and is equal to 1.331. The measured adlayer effective thickness at full coverage was calculated from Equation (11) to be d=0.91±0.09 nm. Considering the specific volume of BSA, the protein concentration on the surface was estimated as $\Gamma_{SPR}=125\pm 13\;\mathrm{ng}\;{\mathrm{cm}}^{-2}$, similar to previous studies [37,49]. It must be stressed that the above estimation of the adlayer thickness and adsorbed protein mass is based on the refractive index of pure protein without considering the presence of coupled solvent molecules in the protein film [37]. If we assume an adlayer with refractive index 1.465, a typical value for hydrated proteins [58,59,60], the calculated values of thickness and protein mass will change considerably (d=1.8 nm, $\Gamma_{SPR}=246\;\mathrm{ng}\;{\mathrm{cm}}^{-2}$).

**Figure 16:**
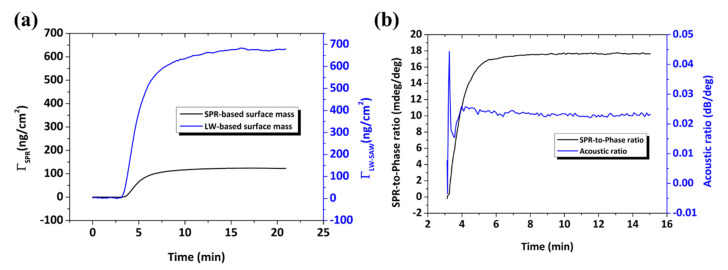
(**a**) Real-time BSA mass uptake estimated by means of phase and SPR response. (**b**) Real-time ratios between SPR-phase and amplitude–phase responses.

The amount of protein on the surface can also be quantified using the phase response and the rigid mass loading phase sensitivity, which is equivalent to the Sauerbrey relation [61]

(15) $$\Gamma_{LW-SAW}\left(\mathrm{ng}\;{\mathrm{cm}}^{-2}\right)=\frac{\Delta \varphi }{\varphi_{0}S_{\Phi }}$$

where $S_{\Phi }=163\;{\mathrm{cm}}^{2}/g$ from Equation (1) and $\varphi_{0}=45000{}^{\circ}$ for the effective length x=0.4 cm. The protein concentration on the surface is estimated to be $\Gamma_{LW-SAW}=668\pm 27\;\mathrm{ng}\;{\mathrm{cm}}^{-2}$, fairly comparable to values obtained by QCM-D analysis [47,51,53,62]. The real-time BSA adsorption on Au in terms of surface mass uptake as detected by means of LW and SPR measurements is presented in Figure 16a. The estimated protein mass uptake with the LW technique is 5.3-fold higher than by estimation with SPR. This difference can be attributed to bound water or water hydrodynamically coupled to the protein layer [60,63].

Complementary to the mass uptake information provided either by the acoustic phase or the SPR response, the amplitude measurement gives information about the energy dissipated during the adsorption process. It is known that the acoustic ratio, i.e., the ratio between amplitude and phase (dB/deg), can be used as a qualitative indicator of the rigidity of the film [59] or to assess the intrinsic viscosity of single biomolecules [2,64]. The temporal variation of the acoustic ratio during BSA adsorption is presented in Figure 16b, from which we obtain a final value of 0.022 ± 0.01 dB/deg. Moreover, the SPR-to-phase ratio (mdeg/deg) can provide a qualitative measure of the hydration of the adsorbed layer. Following the real-time SPR and phase measurements (Figure 16b), the hydration profile is revealed during the entire adsorption process. It is observed that, at low surface coverage, the hydration of the protein layer is higher as expected and is gradually decreasing until the relationship between the two signals is stabilized to a plateau value 17.8 mdeg/deg. The effect is attributed to the presence of water on the surface, detected only by the acoustic measurement while the optical is not affected [60]. 

## 4. Conclusions

In this study, a novel system was engineered to facilitate combined optical and acoustic measurements (SPR/LW-SAW) in a real time fashion on a single surface. For the first time, we obtained the synchronized monitoring of three parameters, i.e., two acoustic, phase and amplitude, and one optical, in a reliable and reproducible way employing a 3D-printed holder and PDMS microfluidic chip allowing for flow-through liquid experiments. The hybrid sensor device was optimized in terms of materials, including the PMMA acoustic waveguide and Au sensing layers. The system was calibrated experimentally by performing glycerol/water and sucrose/water experiments. For the SPR measurements, we determined the conversion factors between the angle of incident light, detector pixel element and refractive index of medium which allow quantitative analysis. The phase and amplitude changes were found to vary linearly with the square root of density–viscosity product upon pure viscous loading, as theoretically expected. Finally, the system was tested by studying the adsorption of protein on Au. The (optical) “dry” and the (acoustic) “wet” masses were obtained allowing for the film hydration to be estimated. Having verified the above-mentioned characteristics, the developed platform could lead to improved insights on biomolecular interactions. Our future aim is to explore the combined instrument to study other biological systems/processes, such as DNA hybridization [65], DNA–protein or RNA–protein interactions [66], lipid particle properties [67], protein–protein interactions [68] and cell attachment at the solid–liquid interface [69]. Data presented in this work are a promising start.
